# Neurorestoratologic Strategies and Mechanisms in the Nervous System

**DOI:** 10.1155/2015/163170

**Published:** 2015-09-29

**Authors:** Lin Chen, Qiang Ao, Hari Shanker Sharma, Aijun Wang, Shiqing Feng

**Affiliations:** ^1^Tsinghua University Yuquan Hospital, 5 Shijingshan Road, Shijingshan District, Beijing 100040, China; ^2^Department of Tissue Engineering, China Medical University, Shenyang 110122, China; ^3^Uppsala University, Uppsala, Sweden; ^4^University of California Davis Medical Center, Surgical Bioengineering Laboratory, Department of Surgery, Sacramento, CA 95817, USA; ^5^Tianjin Medical University, Tianjin, China

Neurorestoratology is an interdisciplinary and frontier discipline that studies neural regeneration, repair, and replacement of damaged components of the nervous system, neuroplasticity, neuroprotection, neuromodulation, neurogenesis, angiogenesis, immunomodulation, and their mechanisms to cause improvement or recovery. Neurorestoratology research is becoming one of the most promising neuroscience fields and a core aspect of translational neuroscience. Neurorestorative therapies include the transplantation of tissue and cells, biomaterials and bioengineering, neuromodulation by electromagnetic stimulation, and pharmaceutical or chemical therapies. Research advancements in the area have been increased tremendously over the past few years, extending from the laboratories to animal experiments and, more recently, to clinical trials.

Last year, we invited investigators to contribute original research as well as review articles that address this field. We encourage manuscripts that will stimulate continuing efforts to understand the mechanisms underlying interaction between environment stressors and neurorestoration in neural trauma, stoke, and neurodegenerative diseases. Today, we are very happy to publish this special issue of this journal.

In this issue, original research and review articles are accepted. The research papers focused on using bone marrow mesenchymal stem cells to treat Alzheimer's disease, examining the effects of lithium on brain inflammatory mediators levels, fever, and mortality in postischemic stroke rats, investigating whether serum lost goodwill target proteome is a potential biomarker of severe traumatic brain injury and if high-caloric diet impaired learning and memory function and the potential mechanisms underlying this process.

The review described the present status of neurorestoratology fields, including the role of ABC transporters in the drug resistance mechanism of intractable epilepsy, bypassing P-glycoprotein drug efflux mechanisms in pharmacoresistant schizophrenia therapy, nerve conduits in the treatment of peripheral nerve injury, and oligodendrocyte precursor cells and biomaterial approaches in spinal cord injury. The papers sample the extraordinary progress that has been made in the field.

Current research suggests that new developments will continue to follow. Particular importance will be given to manuscripts that address the development of novel strategies for therapeutic intervention of these diseases. Our aim is to shed light on important knowledge gaps and to provoke thoughts for further research and the development of therapeutic strategies.

*Lin Chen* *Qiang Ao* *Hari Shanker Sharma* *Aijun Wang* *Shiqing Feng*

